# Radiopharmaceuticals and Fluorescein Sodium Mediated Triple‐Modality Molecular Imaging Allows Precise Image‐Guided Tumor Surgery

**DOI:** 10.1002/advs.201900159

**Published:** 2019-04-24

**Authors:** Sheng Zheng, Zeyu Zhang, Yawei Qu, Xiaojun Zhang, Hongbo Guo, Xiaojing Shi, Meishan Cai, Caiguang Cao, Zhenhua Hu, Haifeng Liu, Jie Tian

**Affiliations:** ^1^ Department of Gastroenterology The Third Medical Centre Chinese PLA General Hospital Beijing 100039 China; ^2^ Department of Gastroenterology Anhui No.2 Provincial People's Hospital Hefei 230041 China; ^3^ School of Life Science and Technology Xidian University Xi'an 710071 China; ^4^ Department of Nuclear Medicine Chinese PLA General Hospital Beijing 100853 China; ^5^ School of Information Sciences and Technology Northwest University Xi'an 710127 China; ^6^ CAS Key Laboratory of Molecular Imaging Beijing Key Laboratory of Molecular Imaging The State Key Laboratory of Management and Control for Complex Systems Institute of Automation Chinese Academy of Sciences University of Chinese Academy of Sciences Beijing 100190 China

**Keywords:** Cerenkov luminescence imaging, Cerenkov radiation energy transfer, confocal laser endomicroscopy, malignant tumors, positron emission computed tomography

## Abstract

Radical resection is the most effective method for malignant tumor treatments. However, conventional imaging cannot fully satisfy the clinical needs of surgical navigation. This study presents a novel triple‐modality positron emission tomography (PET)–Cerenkov radiation energy transfer (CRET)–confocal laser endomicroscopy (CLE) imaging strategy for intraoperative tumor imaging and surgical navigation. Using clinical radiopharmaceuticals and fluorescein sodium (FS), this strategy can accurately detect the tumor and guide the tumor surgery. The FS emission property under Cerenkov radiation excitation is investigated using 2‐deoxy‐2‐^18^F‐fluoroglucose and ^11^C‐choline. Performances of the PET–CRET–CLE imaging and the CRET–CLE image‐guided surgery are evaluated on mouse models. The CRET signal at 8 mm depth is stronger than the Cerenkov luminescence at 1 mm depth in phantoms. In vivo experiments indicate that 0.5 mL kg^−1^ of 10% FS generates the strongest CRET signal, which can be observed immediately after FS injection. A surgical navigation study shows that the tumors are precisely detected and resected using intraoperative CRET–CLE. In summary, a PET–CRET–CLE triple‐modality imaging strategy is developed. This strategy can detect the tumors and precisely guide the tumor resection using clinical pharmaceuticals. This triple‐modality imaging shows high potential in surgical navigation research and clinical translation.

## 1 Introduction

Malignant tumors such as hepatocellular carcinoma (HCC), breast cancer, glioblastoma, and lung cancer are important causes of human death.^1^, ^2^, ^3^ Radical resection is of great significance to the prognosis of these malignant tumors in patients.^4^, ^5^, ^6^ However, tumors are difficult to be completely resected during the surgery since the irregular shape and indistinct margins of the tumors.^7^, ^8^ For example, during the HCC surgery, tumor resection area determined mainly depends on the inspection and palpation of the surgeons, which may lead to omission of residual tumors or unnecessary removal of normal tissues.^9^, ^10^ Inaccuracy of the tumor resection can seriously affect prognosis and life quality of HCC patients.^11^, ^12^ Aiming to improve the HCC resection precision, a wide variety of molecular imaging techniques have been explored to visualize tumor margin and guide the surgery.^13^, ^14^ High‐sensitivity whole body imaging techniques such as positron emission tomography (PET) can provide valuable preoperative diagnostics for HCC patients.^15^ However, position shifting of the internal organs and tissues after the laparotomy causes great difficulties to determine the tumor boundaries during the operation.^16^

In recent years, many optical imaging techniques have been studied for the intraoperative surgical navigation.^10^, ^17^ Indocyanine green (ICG) based fluorescence molecular imaging (FMI) has been widely studied for surgical navigation, which owns the advantages of high sensitivity and high temporal resolution.^18^ However, precise tumor detection with ICG FMI is still hard to achieve, since ICG is a nontargeting probe to tumor and may cause false‐positive detection.^19^ Moreover, at present very few fluorescent probes have been approved by the Food and Drug Administration (FDA) for clinical use.^11^, ^20^ Consequently, the clinical applications of FMI especially in the surgical navigation are still quite limited.

Cerenkov luminescence imaging (CLI) is an emerging optical imaging modality based on the Cerenkov radiation that can be generated by a variety of radionuclides such as ^18^F, ^11^C, ^64^Cu, ^131^I, ^177^Lu, etc., and it has been successfully applied for the image‐guided cancer surgery in small animal models.^21^, ^22^ Compared with FMI, CLI can take advantages of numerous FDA approved targeting radiopharmaceuticals for easy clinical translation, and it also shows high specificity in tumor imaging by using these targeting radioactive probes.^9^, ^23^ Moreover, combining CLI with PET enables radiologists and surgeons to easily “see” the same agent before and during surgery, which may avoid repeated injection of probes and improve the accuracy of image‐guided surgery.^24^ However, long exposure time is necessary for CLI to realize image‐guided surgery because of the Cerenkov luminescence (CL) produced by radiopharmaceuticals is weak.^21^, ^22^, ^23^, ^25^

In our previous study, a novel radiopharmaceuticals excited fluorescence imaging (REFI) technique has been developed to dramatically enhance the CLI signal intensity with short exposure time.^25^, ^26^ Then, REFI guided tumor excision was successfully performed on small animal tumor models.^27^ Cerenkov radiation energy transfer (CRET) is another strategy to strengthen the optical signal intensity, which employs fluorescent materials such as quantum dots.^28^ Unfortunately, most of the mediate materials for REFI and CRET are not suitable for clinical applications due to the potential toxicity. In the way of exploring novel radiation responding materials, clinically available imaging agent fluorescein sodium (FS), which has been commonly used for retinal blood vessels imaging, shows absorption spectra mainly at 465–490 nm and emission spectra peak at 520–530 nm.^29^, ^30^ Considering Cerenkov emission spectra of different radionuclides are mainly in the UV–vis range, therefore, we hypothesize that FS can be excited by Cerenkov photon produced by radiopharmaceuticals and then is further used to improve the CRET performance in surgical navigation.

As a macroscopic imaging modality, CRET can locate tumors in living subjects, but light scattering limits the imaging resolution, which makes CRET difficult to precisely delineate the tumor boundaries.^31^ To overcome this problem, we plan to combine confocal laser endomicroscopy (CLE), which is a new type of microscopic imaging based on the principle of tissue reflectance or tissue fluorescence.^32^ The CLE images are generated to elucidate the cellular architecture and microvascular by applying medical imaging agents like FS, and the imaging resolution can reach the level comparable to traditional histology.^33^ Furthermore, CLE has been demonstrated effective for diagnosing malignancies and identifying different lesion margins, such as the tumors in gastric, esophageal, colorectal, and pancreatic.^34^, ^35^

**Scheme 1:**
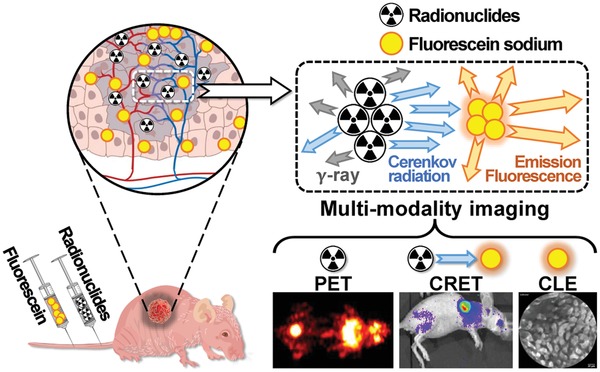
Radiopharmaceutical and fluorescein sodium mediated PET–CRET–CLE triple‐modality imaging for precise tumor surgery.

In this work, we present a novel multimodality imaging approach that attempts to take advantages of PET, CRET, and CLE while avoiding their limitations (**Scheme** 1). Specifically, we first performed whole body PET for preoperative tumor detection after injection of tumor targeting radiopharmaceuticals 2‐deoxy‐2‐^18^F‐fluoroglucose (^18^F‐FDG) and ^11^C‐choline (^11^C‐CHO). Then intraoperative CRET imaging using FS and radiopharmaceuticals as contrast agents was applied to clearly locate the tumors. Based on the CRET imaging results, CLE was conducted to realize precise tumor boundary delineation with high sensitivity and high resolution. To validate the performance of CRET with FS, in vitro experiments were performed followed by in vivo investigation on subcutaneous 4T1 breast cancer mouse models. Then we evaluated the triple‐modality PET–CRET–CLE imaging performance for surgical navigation using orthotropic HCC bearing mouse models. To the best of our knowledge, this is the first study to use radiopharmaceuticals and fluorescein sodium as a triple‐modality surgical navigation strategy, and it allows us to successfully combine PET, CRET, and CLE imaging to achieve precise tumor surgery.

## 2 Results

### 2.1 In Vitro Imaging of 96‐Well Plate Containing Different Samples

**Figure 1:**
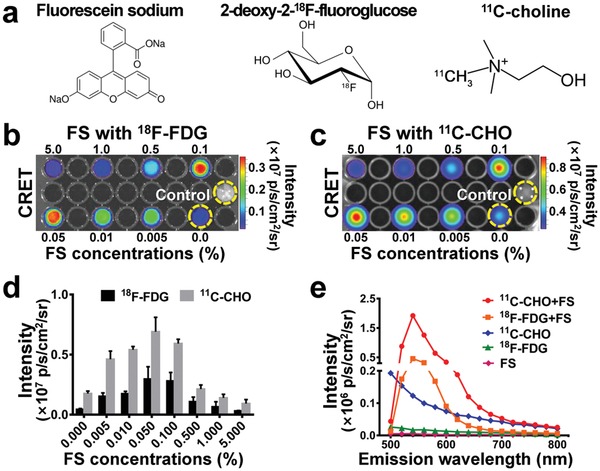
In vitro imaging of 96‐well plate containing different samples. a) The chemical structure of FS, ^18^F‐FDG, and ^11^C‐CHO, respectively. b) CLI of ^18^F‐FDG (100 µCi) and CRET image of the mixture (100 µCi ^18^F‐FDG and FS with various concentrations). c) CLI of ^11^C‐CHO (100 µCi) and CRET image of the mixture (100 µCi ^11^C‐CHO and FS with various concentrations). d) The quantification analysis results of CLI intensity of 100 µCi ^18^F‐FDG and 100 µCi ^11^C‐CHO and CRET signal intensity of the mixture (100 µCi ^18^F‐FDG or ^11^C‐CHO and FS with various concentrations). e) Emission spectra of the mixture (0.05% FS mixed with 100 µCi ^18^F‐FDG or 100 µCi ^11^C‐CHO), ^18^F‐FDG (100 µCi), ^11^C‐CHO (100 µCi), and FS (0.05%).

The chemical structure of the applied FS, ^18^F‐FDG, and ^11^C‐CHO was shown in **Figure** 1a. Experimental results of CLI of ^18^F‐FDG (100 µCi) and CRET of the mixture (100 µCi ^18^F‐FDG and FS with various concentrations) showed that the mixture of ^18^F‐FDG and 0.05% FS had the strongest fluorescence intensity than the other mixtures or ^18^F‐FDG alone (Figure 1b). The quantification analysis results indicated that the CRET signal intensity of the mixture (^18^F‐FDG and 0.05% FS) was ≈6.8‐folds of that of CLI signal intensity of ^18^F‐FDG (Figure 1c, 2.97 ± 0.84 vs 0.44 ± 0.06 × 10^6^ p s^−1^ cm^−2^ sr^−1^). Similar to ^18^F‐FDG mixture, the strongest fluorescence intensity was observed in the mixture of ^11^C‐CHO (100 µCi) and 0.05% FS (Figure 1c). The quantification analysis showed the CRET signal intensity of ^11^C‐CHO and FS mixture was ≈4.1‐folds of that of ^11^C‐CHO CLI signal alone (Figure 1d, 6.9 ± 0.98 vs 1.7 ± 0.17 × 10^6^ p s^−1^ cm^−2^ sr^−1^). Interestingly, the observed fluorescence intensity in the ^11^C‐CHO (and FS) mixture was much stronger than the ^18^F‐FDG (and FS) mixture.

Measurement of emission spectra of the sample (0.05% FS with 100 µCi ^18^F‐FDG or 100 µCi ^11^C‐CHO), ^18^F‐FDG (100 µCi), ^11^C‐CHO (100 µCi), and FS (0.05%) was performed and shown in Figure 1e. It was found that the emission peak was at 540 nm for the two mixtures (^18^F‐FDG or ^11^C‐CHO mixed with FS). The CRET signal intensity began to significantly increase from the wavelength of 500 nm, and exceeded the CLI signal of ^11^C‐CHO at 520 nm (Figure 1e). This observation indicated that the Cerenkov luminescence (less than 500 nm) contributed to the excitation of FS and part of its energy was red‐shifted from blue purple wavelength to the longer wavelength.

### 2.2 Phantom Study for the Comparison of Penetration Depth of CRET and CLT

**Figure 2:**
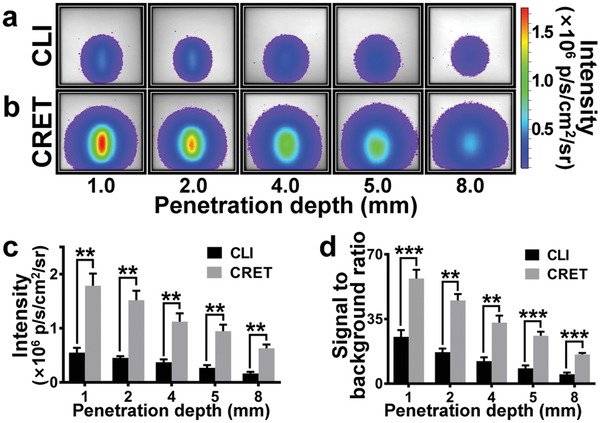
Phantom study for the comparison of penetration depth of CRET and CLI. a) CLI of 100 µCi ^18^F‐FDG. Depth of the source was 1, 2, 4, 5, and 8 mm, respectively. b) CRET of 0.05% FS excited by 100 µCi ^18^F‐FDG in different depths. c) The quantification analysis results of the comparison of the signal intensity of CRET and CLI in different depths. Data are means ± SD. **P ≤ 0.01. d) There was significant difference between the SBR of CLI and CRET imaging in various depths. Data are means ± SD. **P ≤ 0.01, and ***P ≤ 0.001.

The phantom study showed that Cerenkov luminescence intensity of ^18^F‐FDG (100 µCi) decreased with the increase of depth (**Figure** 2a). For the phantom with different source depths, when 0.05% FS (50 µL) was mixed with the ^18^F‐FDG (100 µCi, 50 µL), the CRET signal intensity was obviously stronger than that of ^18^F‐FDG CLI alone (Figure 2a,b). Similar to the CLI, the signal intensity of CRET decreased with the increasing of depth. When the penetration depth was 5 mm, the optical signal intensity of CRET was 3.95 times of that of CLI (0.83 ± 0.09 vs 0.21 ± 0.04 × 10^6^ p s^−1^ cm^−2^ sr^−1^). At the depth of 8 mm, both the CRET and CLI still showed light emitted from the deep source. But CRET clearly showed much higher signal intensity than that of CLI.

Quantification analysis results further indicated that both CLI and CRET signals significantly decreased with the increasing depth of the source. Moreover, CRET signal was much stronger than that of CLI (*P* ≤ 0.01) (Figure 2c). The CRET signal intensity from the ^18^F‐FDG (and 0.05% FS) mixture at 8 mm phantom depth was even stronger than the CLI signal intensity of ^18^F‐FDG at 1 mm phantom depth (0.62 ± 0.06 vs 0.56 ± 0.07 × 10^6^ p s^−1^ cm^−2^ sr^−1^). Obviously adding FS to radiopharmaceuticals (CRET) dramatically improved the light production than them alone (CLI). There was significant difference between the signal to background ratio (SBR) of CLI and CRET imaging in various depths (*P* ≤ 0.01) (Figure 2d).

### 2.3 Dose Effect of FS for In Vivo CRET Imaging

**Figure 3:**
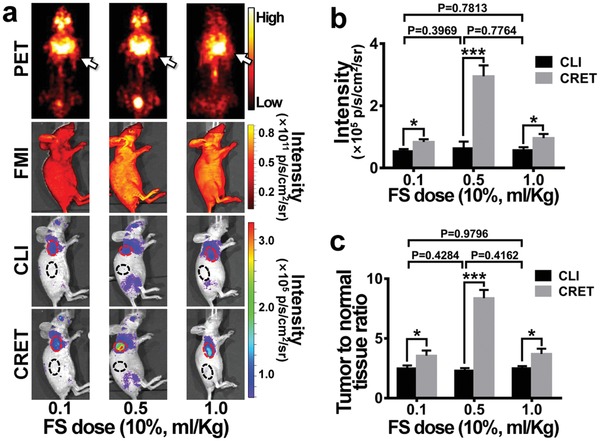
Dose effect of FS for in vivo CRET imaging. a) PET and CLI imaging were performed on three groups of 4T1‐Red‐FLuc tumor‐bearing mice (*n* = 3 per group) after injection of the same activity of ^18^F‐FDG (300 µCi, 100 µL). CRET and fluorescence imaging were performed on the same mice after injection of different doses of FS (10%, 0.1, 0.5, 1.0 mL kg^−1^) of FS. b,c) Comparison of signal intensities and TNR of CLI and CRET. *P ≤ 0.05, **P ≤ 0.01, ***P ≤ 0.001.

PET images of the mice bearing subcutaneous 4T1 breast tumors (three groups, *n* = 3 per group) injected with ^18^F‐FDG (300 µCi, 100 µL) through tail vein did not clearly delineate tumors (**Figure** 3a, the first row). However, CLI of the same mice successfully identified the subcutaneous tumors (Figure 3a, the third row), although the signal of the tumors was very weak and tumor‐to‐normal tissue ratio (TNR) was very low as well. Impressively, after intravenous injection of different doses of FS, CRET imaging results showed that the tumor signals were enhanced obviously and CRET signal reached the strongest when using the FS (10%) dose of 0.5 mL kg^−1^ (Figure 3a, the forth row). It was noted that FMI results showed the fluorescence signals emitted from FS uniformly distributed in the whole mouse and no specific accumulation was observed in the tumor (Figure 3a, the second row).

Three different FS doses were applied to the three groups of subcutaneous 4T1 models. The signal intensity of CRET and CLI had significant difference (Figure 3b, *P* ≤ 0.05) within each group, while no statistical difference of CLI intensity existed between the three groups (*P* > 0.05). Specially for the FS (10%) dose of 0.5 mL kg^−1^, the CRET signal intensity of tumor was 4.7 times of that of CLI (2.95 ± 0.2 vs 0.63 ± 0.13 × 10^5^ p s^−1^ cm^−2^ sr^−1^, *P* ≤ 0.001., Figure 3b). Importantly, CRET imaging also showed much higher TNR than that of CLI (8.4 ± 0.34 vs 2.3 ± 0.12, *P* ≤ 0.001, Figure 3c).

**Figure 4:**
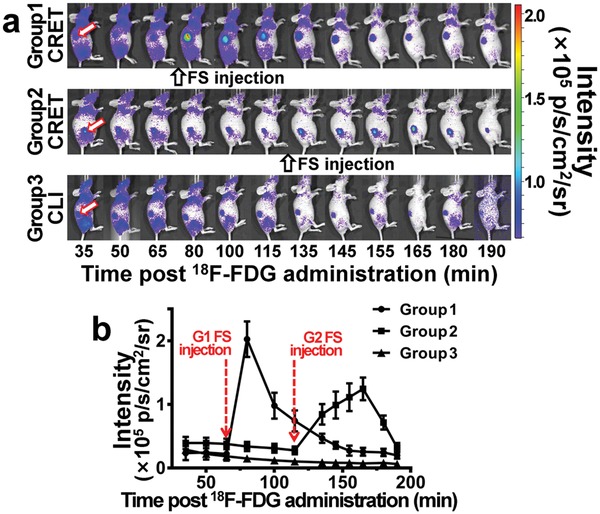
The in vivo validation of FS mediated CRET effect. a) Sequential optical imaging of three groups of 4T1 subcutaneous mouse models injected with the same activity of ^18^F‐FDG (300 µCi, 100 µL). The red arrows indicate the tumor locations. FS of 50 mg kg^−1^ was administrated to group 1 (at 65 min postinjection of ^18^F‐FDG) and group 2 (at 115 min postinjection of ^18^F‐FDG). Group 3 was only injected with ^18^F‐FDG for observing the varying CLI signals. b) Quantitative analysis of the optical signal intensities at tumor sites on the three groups 4T1 subcutaneous mouse models. Red arrows indicate the time points of FS injection.

Furthermore, mice bearing subcutaneous 4T1 presented in the abdomen (three groups, *n* = 3 per group) were injected with ^18^F‐FDG (300 µCi, 100 µL) through the tail‐vein, followed by administrating with FS (10%, 0.5 mL kg^−1^) at different time points postinjection of the radioactive probe. 35 min after the ^18^F‐FDG injection, in vivo CLI was performed and the tumors were localized with low TNR (**Figure** 4a). After the FS administration, the optical signal and the TNR were significantly intensified successively. Mice in the first group received FS administration at 65 min post the ^18^F‐FDG injection, the signal intensity and the TNR were intensified within 15 min. Similar results were observed in the second group, in which the mice received FS administration at 115 min post the ^18^F‐FDG injection, and the signal intensification was seen within 20 min. This observation indicated the FS small molecules fast distributed within the mouse body. After reaching the peak, the CRET intensity was observed gradually decreased owing to the excretion of FS. In the control group without administrating FS, the CLI signal of the tumor was continuously decreasing (Figure 4a, group 3).

The quantitative analysis further demonstrated that the FS based CRET intensity was 3–5 times higher than the CLI intensity (Figure 4b). With the excretion of the whole‐body distributed FS, the CRET signal intensity was decreasing. The observed signal enhancement maintained for nearly 90 min (Figure 4b). From Figure 4, significant improvement of the optical signal intensity was seen in CRET imaging, and the signal enhancing effect was successfully achieved in vivo with FS administration.

### 2.4 Intraoperative CRET Imaging, CLI, and FMI

**Figure 5:**
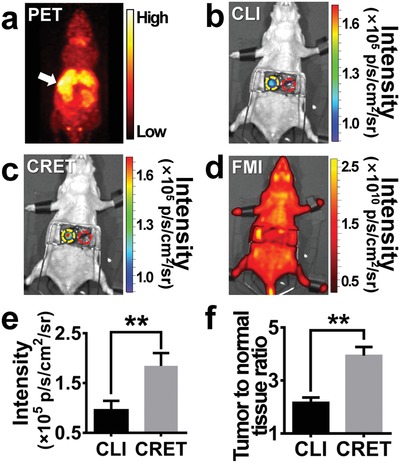
Intraoperative CRET imaging, CLI, and FMI of the orthotropic HCC mouse models (*n* = 3). a) Representative PET image of the mouse at 20 min after injection of ^11^C‐CHO (225 µCi, 100 µL). b) Representative intraoperative CLI of the mouse at 25 min after ^11^C‐CHO injection. c) Intraoperative CRET image acquired 5 min after the FS (50 mg kg^−1^) injection at 30 min of ^11^C‐CHO injection. d) FMI result of the same mouse after the FS injection. e) Comparison of signal intensity of CRET imaging and CLI. Data are mean ± SD. **P ≤ 0.01. f) Comparison of TNRs for CRET and CLI. Data are mean ± SD. **P ≤ 0.01. Yellow circle: regional of interest (ROI) of tumor; red circle: ROI of normal liver tissue.

A typical preoperative PET imaging of the orthotropic HCC mouse models (*n* = 6) injected with ^11^C‐CHO (225 µCi, 100 µL) did not clearly detect HCC in the liver tissues (**Figure** 5a). Nevertheless, interestingly intraoperative CLI distinctly detected the HCC in the right liver lobe (Figure 5b). CRET imaging result also successfully delineated the tumor lesion, in consistence with that of CLI (Figure 5c). It should be noted that the CRET imaging exhibited much higher imaging signal intensity and TNRs than those of CLI (Figure 5e,f; *P* < 0.01). Intraoperative FMI on the same models failed to identify the tumors (Figure 5d).

### 2.5 CRET and CLE Dual Modalities Guided Tumor Resection

**Figure 6:**
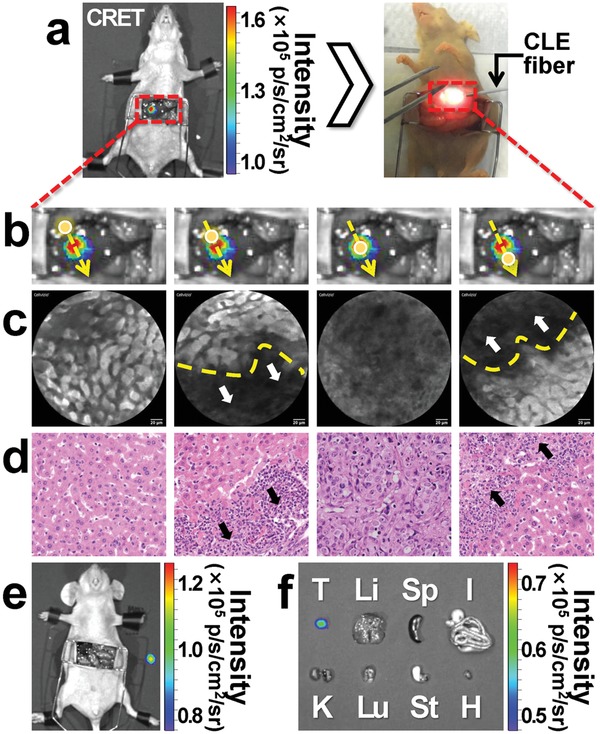
CRET and CLE dual modalities guided tumor resection. a) In vivo CLE imaging was performed on the basis of CRET results (left). The mouse skin turned to yellow color because of the FS injection (right). b) Schematic diagram of CLE detection of tumor margin. The yellow arrows represent the observation path of intraoperative CLE, and the yellow dots represent the exact CLE detecting position correspond to the images shown in (c). c) The intraoperative CLE results at different areas, in which normal liver tissues were shown in high brightness while tumor tissues were in low brightness. The yellow dashed lines indicate the tumor margins, and the white arrows refer to the tumor area in the CLE images. d) 200 times magnified H&E pathological results correspond to the CLE images in (c), from left to right were normal liver, upper tumor margin, tumor tissue, and tumor lower margin, respectively. White arrows in (c) and black arrows in (d) present the same tumor margins in different detections. e,f) CRET imaging results of the resected tumor and the other mouse organs after the image‐guided surgery (T: tumor, Li: liver, Sp: spleen, I: intestine, K: kidney, Lu: lung, St: stomach, H: heart).

In order to further improve the precision of HCC surgical navigation, intraoperative CLE was performed to detect the tumor margin based on CRET imaging results. A typical intraoperative CRET image of the orthotropic HCC mouse models (*n* = 6) clearly delineated HCC in the liver (**Figure** 6a, left). Based on the CRET image, CLE imaging was performed along the yellow line (Figure 6a,b). The yellow dashed arrows represented the observation path of intraoperative CLE, while the round dots indicated the exact CLE detecting positions. Figure 6c showed the acquired CLE results corresponding to each detecting position in Figure 6b. The CLE results visualized the normal liver region, cords of hepatocytes, and sinusoid structure (Figure 6c), in which normal liver tissues were shown in high brightness and the tumor tissues were in low brightness. The yellow dashed lines indicated the tumor margins, and the white arrows referred to the tumor area in the CLE images.

During the entire CLE imaging procedure, the CLE images were significantly distinctive between different imaging areas (Figure 6b,c). When the CLE detector was on the normal liver region, cords of hepatocytes and sinusoid structure were observed by CLE. In contrast to Hematoxylin and Eosin (H&E) staining, the bright cord‐like structure in CLE images was identified as a hepatocyte plate (Figure 6d). When the CLE detector moved forward to the superior margin of the tumor (Figure 6b), the margin between normal and tumor tissue was obviously distinguishable on CLE results. The CLE images of the tumor regions were shown as low intensity, irregular glandular architectural features (Figure 6c). Similarly, CLE imaging also detected lower margin of the tumor, and the tumor tissues were resected according to the CLE tumor margin imaging results. The mice and the resected tumors were then imaged with CRET again. The CRET imaging results of all the mouse organs and the orthotropic HCC are shown in Figure 6e,f. After resection, both in vivo and ex vivo results showed that the optical signals were especially detected in the resected tumor and no signals were observed from the other organs. According to Figure 6c–f, the margin detection results of intraoperative CLE were verified to be consistent with the postoperative H&E pathological results.

## 3 Conclusion and Discussion

Hepatocellular carcinoma, breast cancer, glioblastoma, and lung cancer are common malignant tumors, whereas the radical resection is difficult and the prognosis is poor.^1^, ^2^, ^3^ As an important strategy, using molecular imaging techniques for surgical navigation can provide valuable assistances to improve the surgical performances with high potential for clinical translation.^10^, ^11^, ^12^, ^13^ Herein, we applied FDA approved radiopharmaceuticals and fluorescent contrast agent fluorescein sodium to achieve a novel PET–CRET–CLE triple‐modality surgical navigation regimen, which was further validated in the HCC resection navigation on mice models as a proof of concept study.

PET can provide systemic function and metabolic information of the living subjects, by using various clinical available probes to reflect biological mechanisms of different cancers.^15^ ^18^F‐FDG is a contrast agent for glucose metabolism, and ^11^C‐CHO is for choline metabolism. Both of them are able to achieve precise and targeted tumor detection based on the metabolic differences between the tumor and normal tissues. In this study, the tumors on HCC orthotropic mouse models were detected by high‐sensitivity whole‐body PET using ^11^C‐CHO (Figure 5a). However, PET did not clearly distinguish the size and contour of the tumor because of the resolution is not sufficient.

As a novel promising optical imaging method, Cerenkov luminescence imaging can get access to a large number of clinically available radioactive probes and is well complementary to PET imaging.^22^, ^23^ CLI has also been proven to be an effective method for surgical navigation. In 2010, Holland et al. used CLI for intraoperative navigation and achieved navigational resection of the tumor through the CLI of ^89^Zr‐DFO‐trastuzumab.^36^ In 2013, Madru et al. implemented a dual‐modality navigation imaging of the sentinel lymph node PET/CLI through ^68^Ga‐superparamagnetic iron oxide nanoparticles.^37^ In 2017, Grootendorst et al. achieved clinical application of CLI intraoperative navigation for the first time, and successfully carried out the navigation and resection of breast cancer. The existing outcomes demonstrated the high potential of CLI for intraoperative surgical navigation in clinics.^38^

Aiming to overcome the disadvantage of weak signal intensity of CLI, CRET and REFI were proposed to achieve the enhancement of signal intensity by secondary excitation effect using the fluorescent medium.^25^, ^26^, ^27^ We have conducted intraoperative REFI for surgical navigation and found it to have a better signal‐to‐background ratio than FMI.^27^ However, these studies involved the use of fluorescent nanomaterials such as rare earth nanoparticles, quantum dots, etc. Therefore, these studies raised the concerns on biological toxicity and reduced the clinical feasibility. In the current study, FS is an FDA approved medical fluorescent contrast agent with an excitation peak at 465–490 nm and an emission peak at 520–530 nm. It has been widely used in clinics for retinal blood vessel imaging and CLE imaging. Thus, FS‐mediated CRET imaging can improve the CLI signals intensity with immediate clinical translation capability. In vitro experiments have shown that 0.05% FS induces the strongest enhancement of CLI signals. Employing the CRET effect, optical signals from ^18^F‐FDG (100 µCi) and ^11^C‐CHO (100 µCi) can be strengthened to 6.8 and 4.1 times than their CLI signals, respectively (Figure 1c). CRET with FS can also red‐shift the CLI emission wavelength to near 540 nm (Figure 1d). Furthermore, in the phantom experiments, the CRET strategy can enhance the penetration depth by 7 mm compared to CLI (Figure 2a–c). Improved signal intensity and penetration depth make FS based CRET imaging more attractive for clinical translation than CLI.

During the in vivo experiments, we found that the radiopharmaceutical distribution obtained from PET and CLI results was in high consistence. Then we administrated three different doses of clinical grade 10% FS (0.1, 0.5, 1.0 mL kg^−1^) to validate the CRET performances. Comparing with CLI, the optical signals were significantly enhanced with FS injection in CRET imaging (Figure 3a), and it has been found that 10% FS of 0.5 mL kg^−1^ provides the strongest signal enhancement among the three doses tested. The optical signal intensity in CRET was 4.7 times than CLI, and the SNR was 3.7 times than CLI (Figure 3b,c). Distinction of the optimal FS dose was observed between the in vitro and in vivo CRET studies, which was mainly caused by the dilution effect from blood in the living subjects. From the in vivo results, the nontargeting FS distributed to the whole body with blood flow. In the tumors, FS was excited by the accumulated radiopharmaceuticals to generate strong CRET signals (Figure 4a). The quantitative results indicated that the CRET intensity reached to five times higher than the CLI intensity in vivo (Figure 4b). With the whole‐body distributed FS excreted to a low level, the CRET intensity decreased (Figure 4b). The signal enhancing effect in tumors was observed retained ≈90 min in this study (Figure 4). These results demonstrate that FS is an attractive CRET mediate and owns high potential for future clinical applications.

Furthermore, we performed CLE to combine with the macroscopic imaging techniques (PET and CRET) for achieving high surgical navigation performance. With employment of the fluorescent probe FS, CLE can observe the tumor tissues at the cellular or even subcellular levels in high temporal resolution. FS based in vivo CLE has been studied for digestive tract tumors detection, which showed high potential for intraoperative surgical navigation.^34^

In our triple‐modality HCC molecular imaging surgical navigation method, PET provided a whole‐body scan of the tumor models (Figures 3a and 5a). CLI and CRET can clearly identify the size and contour of the tumors, while CRET owned significantly enhanced signal intensity and SNR at the tumor sites (Figure 5e,f). The results highlighted that CRET can improve the tumor imaging temporal resolution or lower the necessary radiopharmaceutical doses, compared with CLI. Based on the macroscopic CRET tumor imaging results, CLE imaging can well differentiate the normal liver tissues and the tumor tissues (Figure 6b). Then, the tumor margins can be clearly recognized. The tumor margin detection results and the resection performances were confirmed by pathological H&E (Figure 6c,d). The ex vivo CRET and CLE results of the mouse tissues also demonstrated that the HCC was completely resected with help of the triple‐modality surgical navigation method (Figure 6e,f).

In this study, widely used radiopharmaceuticals (^18^F‐FDG and ^11^C‐CHO) and FDA approved fluorescent dye FS were employed to develop a novel in vivo imaging strategy. Radiopharmaceuticals for PET and CLI are functional imaging probes that reflect metabolic properties of the tumors.^39^ FS is a nontargeting fluorescent probe that can be excited by the radiopharmaceuticals. CRET imaging based on the combination of radiopharmaceuticals and FS produces tumor detection results with high specificity, improved signal intensity, and desirable SNR. Meanwhile, CLE imaging using FS reveals the structural information of tissues and can clearly identify the abnormal tumor lesions. Although the PET–CRET–CLE strategy demonstrated desirable in vivo imaging performance, several limitations remain to be overcome. Because FS is a nontargeting fluorescent probe, the specificity of tumor imaging relies on the radiopharmaceutical uptake in tumors. In addition, the fast excretion of FS would raise the difficulty for successive observation. This may also affect the performance of the PET–CRET–CLE to detect tumors when radiopharmaceuticals show poor tumor uptake.^40^ The goal of our future work is to achieve clinical application using FS and radiopharmaceuticals. Image‐guided glioma surgery is one of the potential areas to demonstrate the clinical value of the proposed strategy.

In summary, this study reports a novel triple‐modality PET–CRET–CLE image‐guided HCC surgery strategy, using clinical available molecular probes (radiopharmaceuticals as ^18^F‐FDG and ^11^C‐CHO, and fluorescent probe as fluorescein sodium). This strategy has demonstrated to be effective with high performance and high clinical translation potential. It opens up a new direction for surgical navigation research.

## 4 Experimental Section

*Cell Lines and Reagents*: Well‐differentiated luciferase labeled human HCC cells HepG2‐Red‐Fluc (PerkinElmer, Waltham, MA) were cultured in high‐glucose Dulbecco's modified Eagle's medium (Life Technologies, Carlsbad, CA). Luciferase labeled mouse mammary gland adenocarcinoma cells 4T1‐Red‐FLuc (PerkinElmer, Waltham, MA) were cultured in RPMI 1640 medium (Life Technologies, Carlsbad, CA). Culture media were supplemented with 10% fetal bovine serum (Life Technologies, Carlsbad, CA), 100 U mL^−1^ penicillin, and 0.1 mg mL^−1^ streptomycin. Cells were grown routinely in a monolayer culture at 37 °C in a 5% CO_2_ humidified atmosphere. Clinical grade 10% fluorescein sodium (FLUORESCITE for Intravenous Injection, Alcon Japan Ltd, Tokyo, Japan) was obtained from the General Hospital of Chinese People's Armed Police Forces (Beijing, China). ^18^F‐FDG and ^11^C‐CHO were provided by the Department of Nuclear Medicine, Chinese PLA General Hospital (Beijing, China).

*Subcutaneous and Orthotropic Tumor Models*: 6–8 weeks old female Balb/c athymic nude mice were purchased from Vital River (Beijing, China). The subcutaneous breast cancer 4T1‐Red‐FLuc tumor models (*n* = 18 totally) were established by injecting 1 × 10^6^ 4T1‐Red‐FLuc cells in the right upper flank (*n* = 9) or right lower abdomen (*n* = 9). The orthotropic liver tumor mouse models (*n* = 6) were established by performing a laparotomy in mice under isoflurane gas anesthesia and injecting 5 × 10^6^ HCC HepG2‐Red‐Fluc cells that suspended in 50 µL of Matrigel (Corning, Corning, NY) into the liver lobes. Animal studies were conducted in compliance with the guidelines of the Institutional Animal Care and Use Committee of Chinese PLA General Hospital.

*CRET Effect with FS of Different Concentrations*: Clinical grade 10% FS was diluted in sterile normal saline (0.9% NaCl) to different mass concentrations (5%, 1%, 0.5%, 0.1%, 0.05%, 0.01%, 0.005%) in the same volume of 200 µL. The FS (200 µL) of different concentrations and ^18^F‐FDG (100 µCi, 50 µL) were separately placed into each well of black 96‐well plates. One control sample on every black 96‐well plate was the mixture of normal saline (200 µL) and ^18^F‐FDG (100 µCi, 50 µL). The other control sample was the mixture of FS (0.05%, 200 µL) and normal saline (50 µL). CLI and CRET were performed using IVIS Spectrum in vivo imaging system (PerkinElmer, Waltham, MA), and the imaging parameters were set to: 60 s exposure, 1 f/stop, 4 binning. This experimental process was also conducted with ^11^C‐CHO (100 µCi, 50 µL) aiming to verify the CRET performance in different clinical available radiopharmaceuticals.

In order to explore the emission spectra of CRET imaging, both ^18^F‐FDG and ^11^C‐CHO of 100 µCi (50 µL) were employed to mix with FS (0.05%, 200 µL), respectively. Emission spectra of CRET and CLI using ^18^F‐FDG (100 µCi, 50 µL) and ^11^C‐CHO (100 µCi, 50 µL) were measured by IVIS Spectrum (60 s exposure) with 20 nm band‐pass filters (center wavelength from 500 to 800 nm). FS (0.05%, 250 µL) was used as the control.

All the experiments were replicated at least three times in order to reduce the experimental error. Optical signal intensities were analyzed using the Living Image 3.2 software (PerkinElmer, Waltham, MA) by drawing regions of interest (ROIs).

*CRET Penetration Evaluation*: To compare the penetration depth of CRET and CLI, cubic polyethylene phantoms (*n* = 5) were applied to simulate the biological tissues. For each phantom, both length and width were 40 mm, and a 2.3 mm diameter circular hole was drilled to place the reagents. Distances from the reagent hole walls to the top surfaces were set to 1, 2, 4, 5, and 8 mm, respectively, for simulating different source depths in biological tissues. In the experiment, ^18^F‐FDG (100 µCi, 50 µL) and 50 µL normal saline were mixed and placed in the reagent holes and performed CLI. ^18^F‐FDG (100 µCi, 50 µL) and FS (0.05%, 50 µL) were mixed and placed in the reagent holes to perform CRET. CLI and CRET results are all obtained with 60 s exposure.

*In Vivo PET, CLI, CRET, and FMI of Subcutaneous Mouse Models*: To investigate the optimal dose for injection of the FS, the upper flank subcutaneous 4T1 mouse models (*n* = 3 per group) were randomly divided into three groups and used for the in vivo imaging experiment when the tumor grown for 7 days. ^18^F‐FDG (300 µCi, 100 µL) was injected to each model through the tail vein. After 40 min, small animal PET (PerkinElmer, Waltham, MA) was performed followed by CLI (exposure 300 s, binning 4). Then 10% FS of different doses (0.1, 0.5, 1.0 mL kg^−1^, all in 100 µL) were injected through the tail vein, and 5 min later, CRET imaging (exposure 300 s, binning 4) and FMI (exposure 1 s, binning 4) was conducted successively. The signal intensity of the tumor and the TNR on the CRET and CLI results were analyzed and compared.

To investigate the optimal injection time point of FS, lower abdomen subcutaneous 4T1 mouse models were randomly divided into three groups (*n* = 3 per group) and received the in vivo CRET imaging when the tumor grown for 7 days. ^18^F‐FDG (300 µCi, 100 µL) was administrated to each mouse model through the tail vein. CRET images were acquired (exposure 300 s, binning 4) at twelve time points (35, 50, 65, 80, 100, 115, 135, 145, 155, 165, 180, and 190 min) after the ^18^F‐FDG administration. For the first group, the mice received FS (10%, 0.5 mL kg^−1^, 100 µL) through tail‐vein injection at 65 min after the ^18^F‐FDG injection. For the second group, the same dose of FS was administrated at 115 min after the ^18^F‐FDG injection. The third group was employed as the control without FS injection.

*Intraoperative CRET Imaging and CRET‐CLE Image‐Guided Cancer Surgery of Orthotropic HCC Bearing Mice*: Two weeks after orthotropic inoculation of the HepG2‐Red‐Fluc cells on mice, the HCC models (*n* = 6) were tail‐vein injected with ^11^C‐CHO (225 µCi, 100 µL). 20 min later, PET was performed for preoperative tumor detection. Next, all the HCC bearing mice were anesthetized and received laparotomy followed by intraoperative CLI (exposure 300 s, binning 4). Then CRET imaging (exposure 300 s, binning 4) and fluorescence imaging (exposure 1 s, binning 4) were immediately conducted after tail‐vein injection of FS (10%, 0.5 mL kg^−1^, 100 µL). Optical signal intensities of the tumors and TNRs in CLI and CRET results were quantified and compared.

After the intraoperative CRET imaging and fluorescence imaging, CLE imaging was performed using CellvizioTM (Cellvizio Lab, Mauna Kea Technologies, France) to further precisely delineate the tumor margins. Images were acquired using the UltraMiniO probe with 488 nm excitation that can excite FS to produce fluorescence. During imaging, the CLE detector was slightly touching the surface of tissues for acquisition. Probe characteristics were: diameter 2.6 mm; lateral resolution 1.4 µm; working distance 60 µm; maximal field of view 240 µm; and 8–12 image frames per second. According to the CRET tumor location results, CLE imaging was performed from the normal liver tissues across the tumor tissues to detect the tumor boundaries. Then the tumors were resected based on the CLE tumor boundary detection results. After the tumor resection, CRET results of the mice and the resected tissues were acquired to evaluate the resection quality. Finally, the orthotropic HCC mice (*n* = 6) were sacrificed, and their main organs were collected and received CRET imaging.

*Hematoxylin and Eosin Staining*: After the in vivo experiments, normal liver tissues and resected tumor tissues were fixed in 10% formalin. Formalin and paraffin‐embedded sections (4 mm thickness) were prepared for H&E staining. The slices were observed with light microscope (Leica, Buffalo Grove, IL).

*Statistical Analysis*: The experimental data were presented as mean ± standard deviation. Statistical significance was determined using the student's *t*‐test (Prism v6.0, GraphPad, La Jolla, CA). Differences between groups were considered significantly if *P* ≤ 0.05. Average and standard deviation (SD) were calculated for experiments performed in triplicate. No SD indicated that the relevant measurement was performed only once.

## Conflict of Interest

The authors declare no conflict of interest.
